# Epidemiology and Management of BVDV in Rangeland Beef Breeding Herds in Northern Australia

**DOI:** 10.3390/v12101063

**Published:** 2020-09-23

**Authors:** Michael McGowan, Kieren McCosker, Geoff Fordyce, Peter Kirkland

**Affiliations:** 1 School of Veterinary Science, The University of Queensland, Gatton, QLD 4343, Australia; 2 Department of Primary Industry and Resources, Katherine, NT 0851, Australia; kieren.mccosker@nt.gov.au; 3 Queensland Alliance for Agriculture and Food Innovation, Centre for Animal Science, The University of Queensland, St Lucia, QLD 4072, Australia; g.fordyce@uq.edu.au; 4 Elizabeth Macarthur Agricultural Institute, PMB 4008, Narellan, NSW 2567, Australia; peter.kirkland@dpi.nsw.gov.au

**Keywords:** epidemiology, BVDV, rangeland beef herds, northern Australia

## Abstract

Approximately 60% of Australia’s beef cattle are located in the vast rangelands of northern Australia. Despite the often low stocking densities and extensive management practices of the observed herd, animal prevalence of BVDV infection and typical rates of transmission are similar to those observed in intensively managed herds in southern Australia and elsewhere in the world. A recent large three- to four-year study of factors affecting the reproductive performance of breeding herds in this region found that where there was evidence of widespread and/or recent BVDV infection, the percentage of lactating cows that became pregnant within four months of calving was reduced by 23%, and calf wastage was increased by 9%. BVDV is now considered the second most important endemic disease affecting beef cattle in northern Australia, costing the industry an estimated AUD 50.9 million annually. Although an effective killed vaccine was released in Australia in 2003, the adoption of routine whole herd vaccination by commercial beef farmers has been slow. However, routine testing to identify persistently infected replacement breeding bulls and heifers has been more widely adopted.

## 1. Introduction

Approximately 60% of Australia’s beef cattle are located in northern Australia, which includes the state of Queensland, the Northern Territory, and the northern part of the state of Western Australia [1]. This is a subtropical-tropical region with a characteristic wet and dry season dominated by a summer rainfall pattern. Cattle predominantly graze either native rangeland pastures or those containing introduced tropical grasses and legumes, which all vary considerably in dry matter digestibility and crude protein content according to the season. Approximately 85% of beef cattle in this region contain at least some *Bos indicus* genetics, enabling them to better cope with high environmental temperatures, low quality grazing pastures, and internal and external parasitism, in particular cattle tick (*Rhipicephalus microplus*) and buffalo fly (*Haematobia irritans exigua*) infestations. Median property and paddock size [1] vary considerably across this region (60 to 1250 km^2^ and 419 to 2611 ha, respectively) and stocking rates are typically moderate to low; one adult equivalent (AE) in 5–30 ha, but in some areas, this is as low as 1 AE to 150 ha. Cattle are extensively managed, typically only being mustered (brought in from the paddock and handled through the cattle yards and crush or chute) twice a year, between April to June and August to October, when a number of husbandry and management practices are performed, such as branding and weaning, pregnancy diagnosis, drafting cattle into new management groups, and identification and removal of cull cows and bulls. Notably, this is generally the only time when disease control measures, such as the vaccination of cattle, are conducted. Approximately two-thirds of cow herds in the northern dry tropical rangelands are continuously mated [1], whereas in the more intensively managed southern areas of this region, herds are control mated, typically for periods of four to seven months. Peak calving occurs from October through to January. A consequence of the long mating periods in this region is that at any time there are always some cattle being mated through to six months of gestation, and thus at risk of bovine viral diarrhoea virus (BVDV) induced disease.

In Australia, BVDV was first reported to have been isolated from cases of acute and chronic mucosal disease of cattle in 1964 [2]. Several years later, the findings of the first serological survey of Australian cattle were published [3], and reported a 91% crude prevalence of seropositive cattle sampled from herds located north of the Tropic of Capricorn compared to a prevalence of 54% for those located south of the Tropic. The proportion of seropositive herds was similar, 96% and 84%, respectively. Overall, the estimated prevalence of seropositive herds was 89%, confirming that the national herd was already endemically infected. In 2007 [4], phylogenetic analysis of Australian isolates of BVDV, primarily from persistently infected (PI) cattle collected over a 25-year study, found that 96.3% were BVDV-1c strains, with the remainder being either BVDV-1a (3.1%) or 1b strains (0.3%). No type-2 isolates or Hobi-like (type 3) were identified. Notably, subgenotype 1c has only been reported in Japan, Chile, Argentina, Spain, and South Africa [4]; the first cattle imported into Australia came from South Africa.

It has been estimated [5] that approximately 1% of cattle in the Australian national herd are PI. This is supported by the results of antigen capture ELISA testing of young cattle (9–24 months of age) for export (*n* = 24,035) and health certification (*n* = 13,800) conducted at the Elizabeth MacArthur Agricultural Institute between approximately 2004 and 2010; 1.1% and 1.53% of cattle were confirmed PI, respectively (P. Kirkland pers. comm.). The prevalence of PI cattle amongst breeding-age bulls and heifers in Queensland, where approximately half of Australia’s cattle reside, was 0.35%. This review focuses on updating the knowledge and understanding of the epidemiology and management of BVDV in northern Australia. 

## 2. Prevalence

In the mid-1990s, as part of a structured animal health surveillance programme conducted by the Queensland government, a sample of heifers and cows from 213 Queensland beef herds was bled, and the BVDV seroprevalence was determined using a virus neutralisation test [6]. The overall individual animal seroprevalence was 45%. Despite significant differences between regions with respect to environment and herd management, there was no significant difference between two consecutive years in the percentage of heifer groups and herds, which were estimated to be entirely seronegative (28–41% and 9–13%, respectively). However, herds with 500 or more cattle had a significantly higher likelihood of containing one or more seropositive cattle. In small herds, spontaneous elimination of infection is likely more common because following an outbreak of infection, most cattle become naturally immune and PI cattle have a significantly shortened life expectancy. In larger herds with multiple management groups, typically there are some groups with a high proportion of naïve cattle, with the mixing of cattle from different groups resulting in the ongoing birth of PI cattle. For example, the seroprevalence of seven breeding groups of heifers and cows on a farm in south-east Queensland ranged from 0–80%, with four groups having a very low seroprevalence and one group having a high seroprevalence (Marbach pers. comm.). 

In a four-year (2007–2011) longitudinal study [1] of factors affecting the reproductive performance of 73 commercial beef herds across northern Australia, sera from a cross-sectional sample of cattle in each enrolled breeding group were tested using a BVDV Agar Gel Immunodiffusion test (AGID) [7]. The advantage of using this serological test is that both the prevalence of seropositive cattle and the prevalence of recently infected cattle (those with an AGID test result of three or greater) can be estimated, and test results are not affected by vaccination.

The overall seroprevalence was similar for heifers and cows and between years, varying between 50% and 55%. The median management group seroprevalence was also similar across regions and years. Approximately one in four and one in five heifer and cow groups, respectively, were mostly naïve (<20% seroprevalence), and 3 in 10 and 4 in 10 heifer and cow groups, respectively, were mostly naturally immune (>80% seroprevalence; Table 1). Although the stocking rate is generally low, the typical behaviour of cattle in this hot region of Australia, including the daily close congregation of animals around watering points and nutritional supplementation sites, is likely to encourage the transmission of BVDV. 

The frequency of management groups likely to have experienced an outbreak of BVDV infection (i.e., >30% of AGID test results were ≥3; Table 2) varied between years. In 2009, approximately 3 in 10 and 2 in 10 heifer and cow groups, respectively (Table 2), were likely to have experienced an outbreak of BVDV infection. However, in 2011 only 1 in 10 and 1 in 20 heifer and cow groups, respectively (Table 2), were likely to have experienced an outbreak of BVDV infection. Outbreaks of BVDV infection in groups of heifers are relatively common due to the recognised generally lower age-specific seroprevalence [6]. A serological study of groups of mating age heifers and first-lactation cows in 12 herds in the Northern Territory found that in nearly half of the herds most of the cattle were likely to be susceptible to infection [8]. Further, these estimates of the prevalence of outbreaks of BVDV infection are similar to those derived from collation of laboratory investigations conducted in Queensland and the Northern Territory [9]. Differences between years in the prevalence of outbreaks of infection are likely to reflect the cyclical changes in proportion of naïve and PI cattle in management groups, strongly influenced by herd culling and heifer replacement practices. 

Overall, there is no evidence that the prevalence of infection in beef cattle in northern Australia, either at the herd or animal level, has changed significantly between 1967 and 2009 to 2011.

## 3. Transmission

Reported rates of transmission of BVDV vary considerably depending particularly on the opportunities for close contact between susceptible and PI cattle. When susceptible cattle were placed in close proximity to a PI animal (e.g., kept in a small yard with common water and hay feeder), 60% became infected [10,11] within 24 hours. Under stocking rates of approximately one animal per hectare, transmission rates of 30–60% per month (1–2% per day) are often observed from the temperate beef grazing regions of Australia [10]. However, the birth of a cluster of PI calves can result in approximately 1–4% of susceptible cows becoming infected per day [10]. 

There have been few reports on transmission of BVDV in the extensively managed beef herds of northern Australia. A study of BVDV transmission on two commercial beef properties in central Queensland [12] found that between two days prior to artificial insemination (AI) through to day 51 after AI, 70% and 32% of the seronegative cows and heifers, respectively, seroconverted, but between day 51 and 210, only 17% and 3% of the seronegative cows and heifers, respectively, seroconverted. Thus, during the period in which there was frequent handling to synchronise oestrous and conduct AI and later pregnancy diagnosis (up to day 51), 1% and 0.6% of susceptible cows and heifers became infected per day, respectively; though after this, when cattle were not handled, only 0.1% and 0.02% of susceptible cows and heifers became infected per day, respectively. However, the marked decrease in the rate of transmission after the period of frequent handling cannot be simply explained by the lack of handling alone, but is more likely to have been due to the removal (at the time of pregnancy diagnosis), relocation (planned or inadvertent) or death of some or all of the PI cattle in these herds.

In a north Queensland study [9] of rates of transmission over a two- to six-month period during the dry season in groups (*n* = 199 to 506) of recently weaned calves (3 to 7 months of age), managed in large paddocks (~4000 ha) at stocking rates of one AE per 2–8 ha, 0.5% to 1% of susceptible cattle became infected per day. These rates of transmission are similar to those observed in intensively managed beef herds. Typically in these rangeland herds, calves weaned from different breeding groups are accumulated in large holding yards over one or more weeks, and after processing (castration, dehorning, etc.) graze together in a single paddock, all of which increases the likelihood of transmission of BVDV. A serological study of heifers in herds in the Northern Territory [8] found that although only approximately half the cattle had seroconverted by the time of weaning, by the age of first mating (two to three years of age), virtually all had seroconverted. However, in one herd, there was no evidence of seroconversion at the time of weaning, and by the time of mating, only about 20% had seroconverted. Therefore, although the typical management of young cattle in these herds is likely to encourage the transmission of BVDV, by chance alone in a given year, PI animal(s) may not be present in a paddock of weaned calves, or the PI may associate with only some of the group (particularly in very large paddocks with multiple watering points). This results in a high proportion of cattle being susceptible to infection at the time of their first mating at around two years of age. 

In a serological study [13] of BVDV infection in central Queensland, beef herds showed no significant difference between heifers and young cows in the estimated annual incidence of infection (22% to 29%). A larger study of beef herds across Queensland conducted in 1994–1995 [7] reported similar, albeit lower, estimates of annual incidence of seroconversion (12% to 24%), with again no observed age-related effect. 

## 4. Clinical Presentation

In the first 20 years after its initial detection in Australia, BVDV was considered to primarily cause acute or chronic mucosal disease, with the latter being a much more common presentation. However, fundamental research conducted at the Central Veterinary Laboratory, Glenfield, in the mid-1980s [11] demonstrated that infection of immunologically naïve female cattle around the time of mating resulted in a significantly lower conception rate and an increased rate of late embryonic and early fetal loss without any evidence of clinical disease in the females. Further work by this research group [14] demonstrated that infection of females between 25 to 40 days of gestation resulted in almost all surviving fetuses being born persistently infected. 

Although in northern Australia most mixing of cattle from different paddocks and properties typically occurs around the bi-annual mustering events, problems with maintaining fence security (floods, bushfires, wildlife, and bulls) means that introductions of new cattle to a breeding group can occur at any time. Based on the findings [1] presented in Table 2, about 2 in 10 and 1 in 10 management groups of unvaccinated heifers and cows, respectively, are at risk of experiencing an outbreak of BVDV infection during mating across northern Australia. The most common clinical presentations observed as a consequence of outbreaks of infection in this region are lower than expected pregnancy rates and/or weaning rates, and a higher than expected incidence of weak non-viable calves born and/or ill-thrifty calves at time of weaning. Cases of chronic mucosal disease in yearling and older cattle presenting primarily as marked loss of body condition, and cases of BVDV-induced neurological disease in calves, are only sporadically observed due to paddocks being large and the terrain and vegetation markedly hinder observation of individual cattle.

In these extensively managed herds, the recommended approach to determining whether BVDV infection is likely to have contributed to the observed lower than expected reproductive performance requires, firstly, accounting for the likely impact of the major factors known to affect performance [1], and, secondly, cross-sectional blood sampling of cattle from affected management groups for AGID serology [15]. Multivariable modelling [1] of the impact of the magnitude of BVDV seroprevalence predicted that in management groups with a high seroprevalence (>80% seropositive) at the time of pregnancy diagnosis, the mean percentage of lactating cows that became pregnant within four months of calving was 23% lower (*p* < 0.05; Table 3) than in management groups with a low seroprevalence (<20% seropositive) [1]. In the majority of high seroprevalence management groups, there was evidence from AGID serology of some (>10% of samples tested) recent BVDV infection. Modelling also showed that in management groups that showed evidence of widespread recent BVDV infection (>30% of samples had an AGID test result of ≥3) either at the time of pregnancy diagnosis or weaning, the predicted calf wastage (losses between confirmed pregnancy and weaning) was approximately twice as great (Table 4) as that in groups with evidence of only a low-to-moderate prevalence of recent infection. 

## 5. Economic Impact

In Meat Livestock Australia’s most recent (2015) assessment of the economic cost of endemic disease in the cattle and sheep industries [16], BVDV was considered the second most important disease problem in both southern and northern Australia. Using Australian findings from both experimental and field studies [11,12,14,17,18] of the impact of BVDV infection during mating and gestation, the estimated prevalence of PI cattle [9] and the distribution of prevalence of susceptible cattle in herds in northern Australia [1], it was estimated that a population-level weaning rate would be conservatively reduced by 1–4.5% as a result of between 3% to 7% of heifers/cows being infected each year. Overall the estimated annual economic cost of BVDV infection in the beef industry in northern Australia was AUD 50.9 million.

## 6. Management Strategies

Australian cattle veterinarians and farmers only began to recognise the potential impact of BVDV infection on beef herd performance in the late 1980s, with those involved in the artificial breeding industry being most interested in strategies to prevent outbreaks of infection in groups of cattle undergoing artificial insemination or embryo collection and transfer. However, in contrast to other countries, Australia has only had access to a BVDV vaccine since 2003. Therefore, initial strategies such as blood sample testing of cattle suspected of being PI (e.g., ill-thrifty cattle) and then deliberately exposing selected unmated/non-pregnant females to confirmed PI cattle, or inoculation of these cattle with the live virus either derived from a PI animal [19] or from laboratory culture were used.

Due to recognised differences in strains of BVDV found in Australian cattle and national biosecurity concerns, an Australian killed vaccine was developed by Pfizer Animal Health (Zoetis) using isolates provided by Dr. Peter Kirkland’s laboratory (EMAI). A field evaluation [20] conducted in 2007–2009 demonstrated that in groups of heifers experiencing significant outbreaks of BVDV during mating, the efficacy of the Pestigard® vaccine in preventing infection in progeny of vaccinates was 80% (95% CI, 71–86). Despite the demonstrated efficacy of the vaccine, adoption of routine vaccination of breeding cattle has been relatively slow. A survey [1] of 73 commercial breeding herds located across northern Australia found that in only 6% of herds all breeding cattle were vaccinated, and in 3% of herds, only heifers were vaccinated. Cost of vaccinations and requirement to initially vaccinate cattle twice have been cited as reasons for lack of adoption. The latter issue has been greatly assisted by research, which demonstrated that the interval between the first and second vaccination could be extended to six months, which more readily enables vaccination to be incorporated into routine herd management. Further, with the greater recognition of the impact on beef breeding businesses of an outbreak of BVDV in bull-breeding herds, those using artificial breeding and those using short mating periods are increasingly adopting either vaccination of their heifers only or whole herd vaccination. In some cases, the decision on whether to vaccinate is based on the findings of AGID testing of a cross-section of cattle in each breeding group. 

Perhaps the most common BVDV control measure being used by cattle producers in northern Australia is requiring that all purchased replacement bulls be certified as being not PI. Seedstock producers increasingly test to assess the PI status of their bulls at the time of conducting breeding soundness examinations prior to sale. The availability of a reliable antigen capture by ELISA with high sensitivity and specificity, combined with the convenience of testing either hair or ear-notch samples, has encouraged the adoption of testing to confirm that replacement breeding animals are not PI. 

To support the ongoing efforts of veterinarians and cattle producers to control BVDV in Australia, a technical advisory group has developed comprehensive guidelines defining the approach to investigating and managing BVDV in beef and dairy herds [15]. Further, although there has been some discussion about the possibility of eradicating BVDV from Australian cattle, due to the high prevalence of infection at a herd and animal level, strategic prevention of infection via testing and quarantining of introduced cattle, and vaccination of groups/herds of cattle at risk of an outbreak of infection will be the main approach to BVDV control in Australia for the foreseeable future.

## Figures and Tables

**Table 1:** Distribution of cow and heifer mobs by observed BVDV seroprevalence category [1].

Animal Class	Year	No. of Management Groups	Group Seroprevalence Category *		
			Low	Moderate	High
Heifers	2009	42	31.0%	35.7%	33.3%
	2011	25	24.0%	48.0%	28.0%
Cows ^#^	2009	62	21.0%	38.7%	40.3%
	2011	60	15.0%	50.0%	35.0%

^#^ Some cow groups contained both heifers and cows; * Seroprevalence category defined as: Low, <20%; Moderate, 20–80%; and High, >80% seropositive.

**Table 2:** Distribution of management groups of unvaccinated heifers and cows by prevalence of recent BVDV infection [1].

Animal Class	Year	No. of Management Groups	Frequency of Recent Infection *		
			Low	Moderate	High
Heifers	2009	36	41.7%	30.6%	27.8%
	2011	22	63.6%	27.3%	9.1%
Cows ^#^	2009	57	42.1%	42.1%	15.8%
	2011	54	68.5%	27.8%	3.7%

^#^ Some cow mobs contained both heifers and cows; * Mob prevalence of recent BVDV infection defined as Low: <10%; Moderate: 10–30% and High: >30% AGID test result ≥3.

**Table 3:** Predicted percentage of lactating cows that became pregnant within four months of calving (P4M) by BVDV seroprevalence category [1].

BVDV Seroprevalence *	Mean % P4M	95% Confidence Interval	
		Lower	Upper
Low	57.3 *^A^*	43.8	70.9
Moderate	43.2 *^AB^*	26.2	60.1
High	34.3 *^B^*	17.0	51.6

* Seroprevalence category defined as Low: <20%; Moderate: 20–80% and High: >80% seropositive. Means not sharing a common superscript are significantly different (*p* < 0.05).

**Table 4:** Predicted mean percentage of calf wastage by category of recent BVDV infection [1].

Prevalence * of Recent BVDV	Mean % Calf Wastage	95% Confidence Interval	
		Lower	Upper
Low	11.45 *^A^*	6.51	16.39
Moderate	12.08 *^A^*	7.00	17.16%
High	20.84 *^B^*	12.49	29.19

* Mob prevalence of recent BVDV infection defined as Low: <10%; Moderate: 10–30% and High: >30% AGID test result ≥3. Means not sharing a common superscript are significantly different (*p* < 0.05).
